# Hot Flash Prediction for the Delivery of Just‐In‐Time Interventions

**DOI:** 10.1111/psyp.70056

**Published:** 2025-07-18

**Authors:** Nader Naghavi, Thomas Cook, Ryan Turner, Sofiya Shreyer, Katherine Colfer, Sonja Billes, Matthew Smith, Michael Busa

**Affiliations:** ^1^ Department of Kinesiology University of Massachusetts Amherst Amherst Massachusetts USA; ^2^ Embr Labs Boston Massachusetts USA; ^3^ Institute for Applied Life Sciences, University of Massachusetts Amherst Amherst Massachusetts USA; ^4^ Department of Mathematics and Statistics University of Massachusetts Amherst Massachusetts USA; ^5^ College of Engineering University of Massachusetts Amherst Massachusetts USA; ^6^ Department of Anthropology University of Massachusetts Amherst Massachusetts USA; ^7^ Department of Medicine, University of Massachusetts Chan Medical School Worcester Massachusetts USA

## Abstract

During menopause, the majority of women experience hot flashes (HF) that have a significant negative impact on sleep and quality of life. Current HF therapies are either ineffective or associated with unacceptable side effects. Digital health technologies offer a novel opportunity to fill this treatment gap with just‐in‐time thermal interventions through wearable devices. Thermal interventions have shown promise in reducing the negative impact of HFs. We hypothesized that HF event onsets can be accurately and reliably predicted from physiological signals prior to a person's perception of the events. This study investigated the feasibility of using skin conductance (SC) to predict the onset of HF events before they are subjectively perceived. 62 women who were experiencing HFs and self‐reported being in peri‐ or postmenopause were recruited. Data collection consisted of three remotely conducted 48‐h sessions. During each session, SC from the lateral torso was measured continuously and participants logged the precise timing of each perceived HF event onset. We developed new features to identify characteristics of SC signals before HFs were perceived. The best performing model trained with these features identified 82% of HF events on average 17 s before the onset with less than 2% false‐positive rate. Among the identified events, the model predicted 69% of HF events before onset. This study demonstrates the feasibility of predicting HF event onsets before subjective perception. Future studies should investigate both multimodal prediction as well as user acceptance and effectiveness of just‐in‐time thermal interventions.

## Abstract 2

Impact Statement

This study is the first to demonstrate that hot flashes during menopause can be accurately predicted using skinductance signals, enabling detection an average of 17 s before subjective awareness. This breakthrough opens the door to non‐pharmocological, hormone‐free intervention systems that could help alleviate symptoms and improve the quality of life for millions of individuals affected by menopause.

## 1 Introduction

A hot flash (or hot flush, HF), a type of vasomotor symptom, is characterized by the sensation of intense heat as well as sweating, flushing, and chills (Archer et al. 2011; Freedman 1989). HFs may occur at any time of day or night and may last between 1 and 60 min (Forouzanfar et al. 2018). HFs can be idiopathic or triggered by external stimuli such as embarrassment, stress, increased ambient temperature, warm drinks, caffeine, and alcohol (Archer et al. 2011). Approximately 80% of women experience HFs during menopause, and many women experience frequent HFs for 7 years or more (Avis et al. 2015; Gold et al. 2006). HFs can have a profound negative impact on daytime functioning, productivity, mood, sleep, and quality of life (El Khoudary et al. 2019; Nappi et al. 2021; Shifren et al. 2014; Whiteley et al. 2013), as well as long‐term health and longevity (Monteleone et al. 2018). Treatment options for women who experience bothersome HFs usually consist of hormone therapy with few FDA‐approved nonhormonal medications, including the recently approved fezolinetant (Faubion et al. 2022; Gonzalez‐Garcia and Lopez 2023; Santoro et al. 2021). For individuals with medical contraindications or who do not want to take medication, HF management consists of behavioral strategies, for example, cooling, dressing in layers, and avoiding triggers.

Historically, HF event have been objectively characterized by a sudden increase in skin conductivity due to the sweat response, which tails off gradually due to decreased sweat and evaporation (Freedman 2014). Physiologic measures of HFs enable objective HF assessment, which is useful in evaluating HF mechanisms and the impact of treatment (Sturdee et al. 2017). The most common method for physiologic HF identification relies on the rapid change in skin conductance (SC), or electrodermal activity (EDA), defined as a change of at least 2 μmho over 30 s (Freedman 1989, 2014; Hanisch et al. 2007). The utility of the conventional 2μmho/30 s criterion is limited by low sensitivity and more sophisticated approaches have improved the identification of HFs (Thurston et al. 2009). Examples include Support Vector Machine (SVM) models, which rely on pattern recognition to eliminate artifacts (Thurston et al. 2011, 2009), and algorithms that incorporate the change in sternal SC with heart rate patterns (Forouzanfar et al. 2018). Notably, these methods rely on retrospective processing of physiologic data to detect the occurrence of past HFs as a passive monitoring tool.

The authors hypothesize that real‐time “prediction” of HF events (identification of events before subjective perception of the event onset) will facilitate automatic application of interventions before HFs are perceived, potentially reducing the severity and bothersomeness of the symptoms. However, to test this hypothesis, first the feasibility of prediction of HFs needs to be investigated. To the best of the authors' knowledge, no HF identification methods have been described that can predict HF event onsets before the subjective perception of them. The feasibility of symptom “prediction” before subjective perception (as opposed to “detection” after subjective perception) has been explored in other domains before (Naghavi and Wade 2022). The utility of such a measure would allow for the potential of deploying an automatic just‐in‐time HF intervention before the individual is even aware of a pending HF event. Such an intervention may now be possible with advances in digital health technology. For example, the Embr Wave wearable thermal device (Embr Labs, Boston, MA) applies low intensity dynamic cooling to the inside of the wrist (Wang et al. 2020) that improves HF control and sleep in midlife women (Composto et al. 2021; Wang et al. 2020) and may also reduce HF burden in men with prostate cancer (Peeke et al. 2024). Currently, there is no technology in the market that can automatically and accurately detect a HF event and automatically deliver a cooling intervention. Predictive algorithms may enable rapid delivery of such interventions earlier in the course of a HF, which may provide meaningful relief automatically and result in reductions in the negative impact of HFs on daily activities and sleep.

**FIGURE 1:**
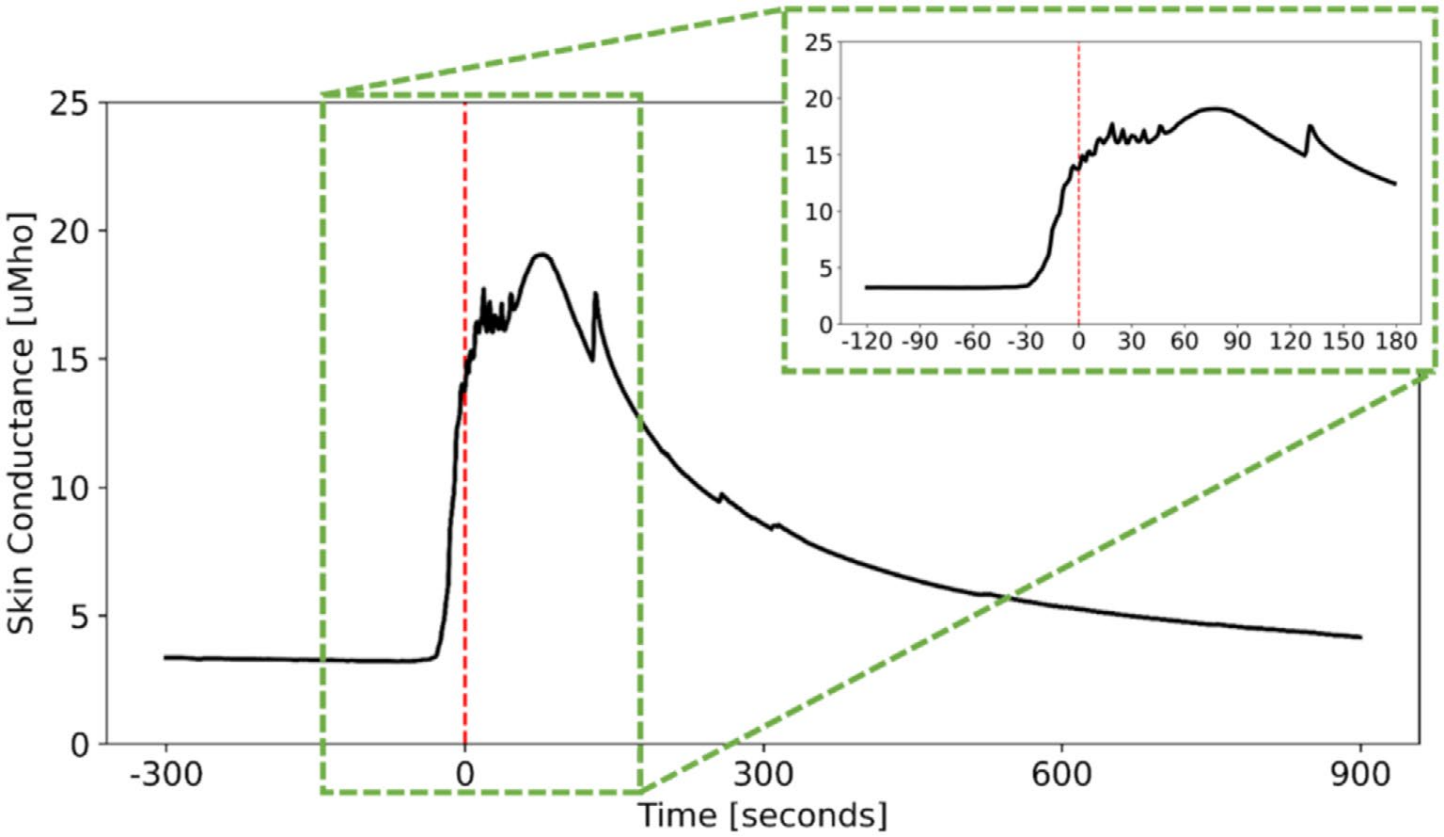
Skin conductance in response to a HF event (*t* = 0 indicated by the red dashed line is the perceived event onset).

This study aimed to develop methods that predict HF event onset before a person subjectively perceives them. Figure 1 shows SC signal change from baseline in response to a HF event. The red dotted vertical line illustrates the timing of the perceived onset of the event. However, the SC signal rises a few seconds before the subjective perception. We hypothesized that analytical features could be established that would allow for the sensitive, early detection of the rising edge in the SC signal prior to subjective HF event onsets. Thus, the methods developed in this study are focused on identifying early characteristics of SC response in order to improve prediction of HF events.

## 2 Methods

### 2.1 Protocol

The protocol (#2084) was approved by the University of Massachusetts Amherst Institutional Review Board. All participants provided written informed consent and were informed that their deidentified data would be published. Eligible study participants were 45 to 65 years of age, in peri‐ or postmenopause (self‐reported irregular periods or no longer menstruating), and self‐reported experiencing HFs at least daily. Participants were excluded if they were taking hormone replacement therapy, hormonal birth control, SSRIs or SNRIs specifically prescribed for HF management, clonidine, gabapentin, pregabalin, antispasmodic oxybutynin, or black cohosh, or were using an Embr Wave device for hot flash management.

Data collection took place from January to October 2021. Study participation consisted of three remotely conducted sessions. A data collection kit was delivered to participants' homes before each session and collected after each session. Each data collection session began with a video call (so study staff could assist participants with donning physiological monitors) followed by the 48‐h ambulatory data collection period. Study staff were available as needed to assist with study procedures and answer questions. Participants were instructed to wear all monitors continuously for each 48‐h session (participants could remove monitors two times during each monitoring period so they could bathe) and to maintain their standard routine and activity levels. Participants were also instructed to record the occurance and timing of HF events as soon as they were perceived using either the provided digital event markers (GENEActiv) or paper logs.

During each data collection session, participants wore an accelerometer on the preferred wrist (GENEActiv, Activinsights), an accelerometer on the nondominant wrist (GT9X LINK, ActiGraph), an ECG sensor below the sternum (EcgMove4, movisens), an EDA sensor on the right lateral torso (EdaMove4, movisens), a temperature monitor (CORE Body Temperature Monitor, GreenTEG) on the left lateral torso, and a HF monitor with one SC electrode on the sternum and one additional electrode on where each participant experienced the most intense heat sensation (3991/1‐SCL Biolog, UFI). Physiological monitors (except BioLog) were synced to the laboratory's network time protocol (NTP) server before delivery to participants' homes. Date and time were set on Biolog by the participant during a call with a study staff member, from the time displayed on the ActiGraph. The raw data were stored locally on each monitor and were downloaded to a secure lab server after each data collection session.

The data collection was part of a larger protocol; thus, multiple monitors were used to collect different physiological data types. This study only included SC data from movisens EDAMove4. Times of perceived HF events were marked digitally via GENEActiv and/or UFI 3991x1‐SCL BioLog, or written in a paper diary if digital marking was not possible.

### 2.2 Participants

The study enrolled 62 participants. Of these, nine withdrew from the study due to equipment discomfort (*n* = 8) and schedule conflict (*n* = 1). Data reported here are from the 53 participants who completed the study. Mean (SD) age was 54.5 (4.3) years. Self‐report race/ethnicity was: 98% White, 6% Hispanic, Latino, or Spanish origin, 4% Asian, 2% Black or African American, and 2% American Indian or Alaska Native.

### 2.3 Data Preparation

#### 2.3.1 Data Quality Checks

A data expert (NN) visually inspected SC data and removed data that could not be used for analysis: when participants reported they were not wearing the device (e.g., during bathing), low data quality due to misplaced electrodes (visual observation of high frequency noise in the signal, or flat and very low signal values), or saturated signal (due to skin moisture after shower).

#### 2.3.2 Expert‐Identified HF Events

Not all HF events may have been logged by participants; for example, participants may have been occupied (e.g., driving), sleeping, or unsure if they had a HF event. Also, the paper diary included estimated times for events that were missing from the digital event logs. To address inconsistencies in HF marker timing and potential missing events, two HF experts visually inspected the data. The first expert (NN) marked potential HF events indicated by SC signal patterns (a spike followed by gradual decay). The second expert (SS) visually inspected the SC signals, approved or rejected all perceived or expert‐identified HF events by the first expert, adjusted perceived HF onset timestamps if the perceived HF event was marked more than 1 min after the peak in SC signal (to address potential delay in subjective HF events), and marked prolonged nighttime HF events (termed “night sweats”), which were excluded from the analysis. Perceived HF events were defined as typical (a spike in SC followed by a gradual decline) and atypical (other SC patterns, possibly attributed to variations in HF physiology, errors in sensor placement, or motion artifacts). Perceived HF markers that were not accompanied by a change in SC were removed. This study used the expert‐identified events as the “gold reference” for model training and testing.

### 2.4 Train and Test Data

To investigate the model performance and generalization to new subjects, a group of participants was set aside for testing (Test Set) and the models were trained using the rest of the data. The Test Set included an equal number of participants with typical and atypical SC patterns during HF events. The selection for the Test Set was based on visual observation of the data by the first expert (NN).

#### 2.4.1 Moving Window

Starting from the first timestamp in the data, each 48‐h session was segmented into “windows” of specified lengths. The window length was a parameter that was optimized for each method (explained in “Feature Extraction”) to achieve the highest performance (“Performance Measures”). A step of 5 s was used to create overlapping windows.

#### 2.4.2 Data Labeling

Each window was labeled based on the presence of an expert‐identified HF event. As this study was aimed to test the feasibility of predicting HF event onsets, a three‐class labeling approach was used in train and test data in order to focus the models on the HF onset. Using a three‐class approach enables cost‐sensitive classification; that is, to further penalize the models for not identifying HF event onsets. A higher cost for not predicting an HF onset (missing a pre‐HF episode) compared to a lower cost for not detecting an HF onset (missing a post‐HF episode) in the train set may potentially push the model boundaries to enable more HF prediction. This approach also enables the trained models to provide information about the state of the HF, which could be used to tailor the HF intervention.

We took a different approach for labeling Train and Test sets. For the Train set, a wide period of time around each HF event onset was included to address potential delays in HF event onsets and to balance the class ratio in the train set. The Test set included a smaller period of time around each HF onset.

#### 2.4.3 Train Dataset and Labels

**FIGURE 2:**
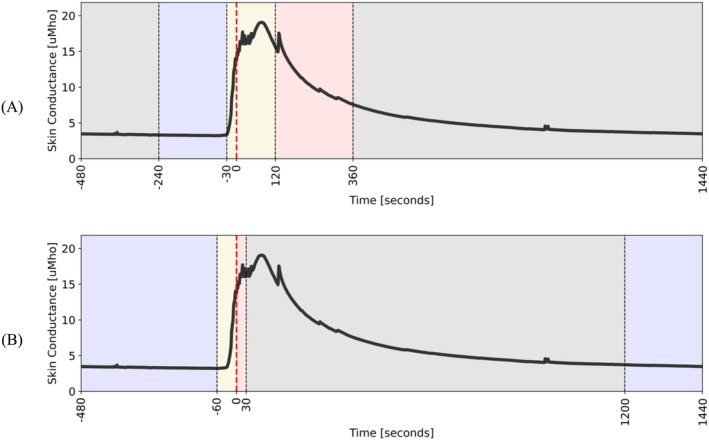
Train (A) and Test (B) data labeling approach. Time is relative to the expert‐identified HF inset (*t* = 0 is the HF event onset), blue: Class 0, yellow: Class 1, red: Class 2, gray: Excluded.

Figure 2A illustrates the labeling approach for the Train dataset:

– Class 0 (non‐HF): Class 0 windows included SC data from before HF event onset. A window was labeled as Class 0 if the last timestamp in the window was within 240 to 30 s before HF onset.
– Class 1 (HF Occurrence): Class 1 windows included SC data from before and after HF event onset; a period that includes the spike in SC signal. A window was labeled Class 1 if the last timestamp in the window was within 30 s before and 120 s after a HF onset. A sensitivity analysis was conducted to identify the optimal pre‐ and post‐HF onset periods to achieve the highest performance.
– Class 2 (HF Persistence): Class 2 windows included SC data from late HF events, the portion of a HF event where physiological indicators return to baseline. A window was labeled Class 2 if the last timestamp in the window was within 120 to 240 s after HF onset.

The rest of the data (gray period in Figure 2A) was excluded from the analysis to improve class balance and reduce bias for non‐HF classification.

Class 0 also included data that were identified by the first expert as potential HFs and rejected by the second expert (240 s before and after event onsets). To improve model performance, through the reduction of false positives, some non‐HF periods that included HF‐like signatures were included.

#### 2.4.4 Test Dataset and Labels

Figure 2B illustrates the labeling approach for the Test dataset:

– Class 0 (non‐HF): Class 0 windows included SC data from before and long after HF event onset. A window was labeled as Class 0 if the last timestamp in the window was more than 60 s before or more than 20 min after a HF onset.
– Class 1 (pre‐HF): Class 1 windows included SC data from immediately before HF event onset. A window was labeled as Class 1 if the last timestamp in the window was within 60 s before and up to HF onset. This period was selected with the hypothesis that prediction of an event 60 s before the onset would provide sufficient time for effective just‐in‐time interventions.
– Class 2 (post‐HF): Class 2 windows included SC data from immediately after HF event onset. A window was labeled as Class 2 if the last timestamp in the window was within HF onset and 30 s after HF onset.

Data from 30 s to 20 min after a HF onset was excluded.

### 2.5 Feature Extraction

Seven novel features were developed to identify the early characteristics of SC changes prior to and immediately after subjective perception of HF event onsets. Raw SC data were collected at 32 Hz and were reduced to 1 Hz using a moving 1‐s mean filter to remove noise.

A 5‐s sliding window was used to extract features. A sensitivity analysis was conducted to identify the optimal window lengths (30, 45, and 60 s) and step lengths (2 and 5 s) for each feature.

#### 2.5.1 Median Subtracted Integral

**FIGURE 3:**
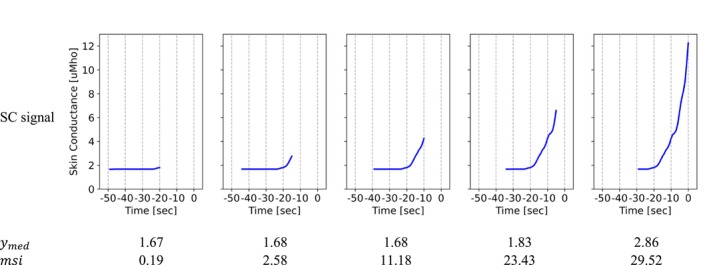
Sliding windows with *msi* feature values for a pre‐HF period. The horizontal axis is the time with respect to the HF onset (*t* = 0 indicates the event onset).

The median subtracted integral (msi) feature was calculated as follows:

$$\mathrm{msi}=\int \left(y-y_{\mathrm{med}}\right)\mathrm{dx}$$

where $y_{\mathrm{med}}$ is the median of the SC signal within each window. A window length of 30 s with a step length of 5 s were identified through a sensitivity analysis for this feature. Figure 3 shows the *msi* values for five successive windows before a HF onset. Although the median value does not change in these five windows, the msi values increase as the window moves forward and contains higher SC values that correspond with the upcoming HF onset (moving from left to right in Figure 3). This feature correlates with third quartile values, but is more sensitive to rapid changes in the SC signal.

#### 2.5.2 Curvefit Family

The curvefit family of features were extracted to estimate the rate of change from a fitted biexponential function on the SC signal. The biexponential equation used to estimate the SC signal was in the form of $y_{c}=A\;e^{Bx^{2}+\mathrm{Cx}}$ where $x\in \left[0,1\right]$ is a list with the same length as the SC signal in each window (x=0 and x=1 represent the first and last data points in the signal, respectively). The biexponential function was then fit on the normalized SC ($y_{n}=y-y_{\min}$) values. The family of curvefit features were calculated as below:

$$d1_{\min}=\min\left({y_{c}}^{\prime }\right)$$

$$d1_{\max}=\max\left({y_{c}}^{\prime }\right)$$

$$d2_{\min}=\min\left({y_{c}}^{\prime \prime }\right)$$

$$d2_{\max}=\max\left({y_{c}}^{\prime \prime }\right)$$

$$d1d2_{\min}=\min\left({y_{c}}^{\prime }\times {y_{c}}^{\prime \prime }\right)$$

$$d1d2_{\max}=\max\left({y_{c}}^{\prime }\times {y_{c}}^{\prime \prime }\right)$$

where ${y_{c}}^{\prime }$ and ${y_{c}}^{\prime \prime }$ are the first and second derivatives of the estimated signal, respectively. A window length of 60 s with a step length of 5 s were identified through a sensitivity analysis for these features.

**FIGURE 4:**
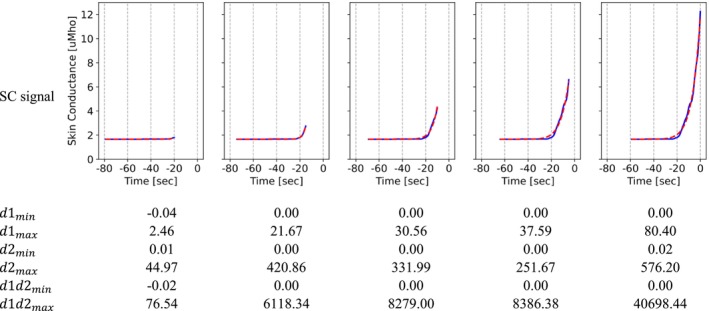
Sliding windows of *curvefit* feature values for a pre‐HF period. The horizontal axis is the time with respect to the HF onset (*t* = 0 indicates the event onset).

Figure 4 shows how the curve fit feature values change before an HF onset. The solid and dashed lines are the raw SC and estimated SC values, respectively. In these plots, the features were calculated every 5 s (moving windows with steps of 5 s) using the last 60 s of the SC signal (moving windows of 60 s long).

### 2.6 Performance Measures

In this study, Class 1 and 2 windows were separated to explore cost‐sensitive classification and penalize models for missing pre‐HF and post‐HF intervals. However, as both Class 1 and Class 2 identify the occurrence of a HF, these two classes were combined into a HF Class for the performance analysis (Table 1).

**TABLE 1:** Confusion matrix for HF identification (Class 1 and 2 are combined into a HF Class in the Test dataset).

		True labels	
		HF (Classes 1 and 2)	Non‐HF (Class 0)
Classification labels	HF (Classes 1 and 2)	TP	FP
	Non‐HF (Class 0)	FN	TN

The following performance measures were calculated for each 48‐h session of the test set. Model performance was evaluated by calculating the mean of each measure for all sessions.

– Sensitivity (Sens): The ability of the model to correctly identify HF among all HF windows: TP/[TP + FN]
– Specificity (Spec): The ability of the model to correctly idenitfy non‐HF among all non‐HF windows: TN/[TN + FP]
– Predictivity* (Pred): The ability of the model to correctly identify HF amon all Class 1 windows.
– Positive Predictive Value (PPV): The probablility that an identified HF window accurately identifies the presence of a HF: TP/[TP + FP]
– Negative Predictive Value (NPV): The probability that an identified non‐HF window accurately idenitifies the presence of non‐HF: TN/[TN + FN]
– F1‐Score (F1): The harmonic mean of PPV and Sens (2TP/[2TP + FP + FN])
– Prediction Rate* (PR): number of correctly predicted HF events within 60 s before the event onset divided by the total number of events per each 48‐h session
– Identification Rate* (IR): number of correctly identified HF events including “predicted” within 60 s before and “detected” within 30 s after the event onset, divided by the total number of events per each 48‐h session
– Identification Latency* (IL): Average latency (in seconds) in HF event onset identification. Negative values indicate HF identification prior to HF marker (prediction). Positive values indicate HF identification after HF marker (detection).
  * indicates novel criteria developed in this study.

### 2.7 Model Training and Testing

Seven univariate logistic regression models were trained with the new features (“Feature Extraction”). Logistic regression models from the Scikit‐learn package (Pedregosa et al. 2011) with L2 penalty, saga solver, 10,000 maximum number of iterations, and a provided random state (*n* = 10) for repeatability of the results were used for training.

Feature values from train and test sets were scaled following the steps below:

– Each feature in the train set was visually inspected, using histograms, for data normality. A nonlinear function was applied to each feature to convert train data into a normal distribution. The same function was then applied to the same feature in the test set.
– From the train set, the median was removed, and the data were scaled to unit interquartile range (IQR, range between the 1st and 3rd quartiles). The feature in the test set was then also scaled using the same median and IQR from the train set.
– An arctan function was applied to both the train and test sets to further limit the range.

The training set had both the mean removed, and the data were scaled to unit variance. The test set was then scaled using the mean from the training set mean and standard deviation from the training set.

Models were trained using the data in the train set. The trained models were then tested on data from the test participants who were not included in the train set. Each test participant included three visits. The performance was evaluated on all visits from participants with typical SC signal, all visits from participants with atypical SC signal, and all visits from both groups. As the features introduced in this study were designed to measure the rate of change in the rising side of the SC spike, the features were expected to be less sensitive in participants with atypical SC signal. We carefully identified these participants and included them in the test set to ensure that the results are not skewed by participants with typical SC signal. This informs the expected variations in model performance in real‐world scenarios. If the test set was randomly selected (like what is normally done in classification studies) we would not be able to make such a comparison between the two categories.

## 3 Results

### 3.1 Train and Test Data

From the 53 participants who completed 3 visits, 10 participants were set aside for the Test Set, including 5 participants with typical HF patterns and 5 with atypical patterns. The models were trained using the remaining data from 43 participants.

### 3.2 HF Event Markers

Overall, 120.5 ± 28.9 (mean ± SD) hours of data was included in the analysis for each participant. Over the three 48‐h visits for each participant, 65.9 ± 36.9 HF events were marked by the expert and included in the analysis. The participants marked 28.9 ± 26.0 events, of which 4.3 ± 5.0 events (16.3% ± 15.2%) were not confirmed by the expert. In this study, 65.1% ± 22.6% of expert‐identified events were not accompanied by a perceived event. Other studies have reported that participants marked approximately 40% of objective hot flashes, with more subjective hot flashes reported during the daytime than during sleep (Carpenter et al. 2004; Otte et al. 2009).

### 3.3 Class Ratio

Table 2 shows the sample size and class ratio (number of samples in a class divided by the total number of samples) for training and testing data sets. While the train set included only intervals before and after HF events, the test data from each participant included the entire 48‐h from each session. In the train set, the Class 1 ratio is lower compared with the other two classes due to the shorter temporal window. In the test set, the majority of data belongs to Class 0 due to shorter HF intervals compared to non‐HF.

**TABLE 2:** Number of samples and class ratio in train and test data.

	Number of samples	Class 0 ratio	Class 1 ratio	Class 2 ratio
Train Data	348,589	41%	21%	38%
Test Data	695,037	97%	2%	1%

### 3.4 Participants With Typical and Atypical HF Patterns

**FIGURE 5:**
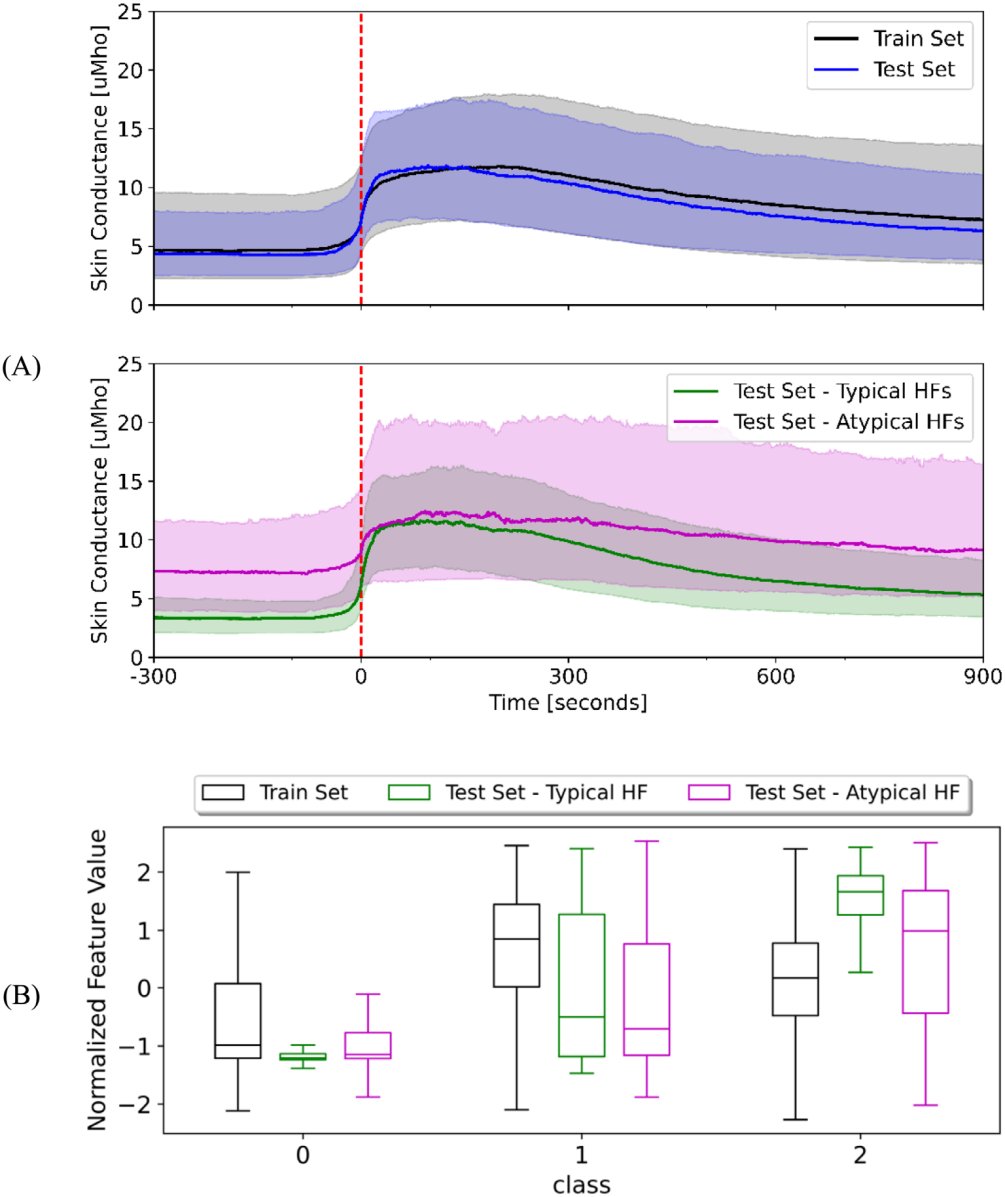
(A) Median and interquartile range of skin conductance signal in response to a HF event. The horizontal axis is the time with respect to the HF onset (*t* = 0 indicates the event onset). (B) Feature values (d2‐max) in the train set and test sets with typical and atypical HF patterns.

Figure 5 shows the median and IQR of SC signal before and after HF events for train and test sets. The signal pattern and range are similar for train and test data, suggesting that the data used for model training and testing come from a similar distribution. The test data was formed by two groups of participants: with mostly typical HF patterns (test set—typical HF) and with lots of atypical HF patterns (test set—atypical HF). The pattern in the typical HF group is similar to the train set; however, the atypical HF group has a slightly different pattern with a higher SC baseline amplitude before HF onset and a smaller change (and, thus, smaller rate of change) after HF onset. Figure 5 also compares values for one feature between the train set and two groups of test sets and shows that the median of feature values in Class 1 (associated with pre‐HF) is lower in the test set with atypical HFs, which suggests lower capability of the model in distinguishing between non‐HF and pre‐HF, that is, predicting HF event onsets.

### 3.5 Detection Versus Prediction

**FIGURE 6:**
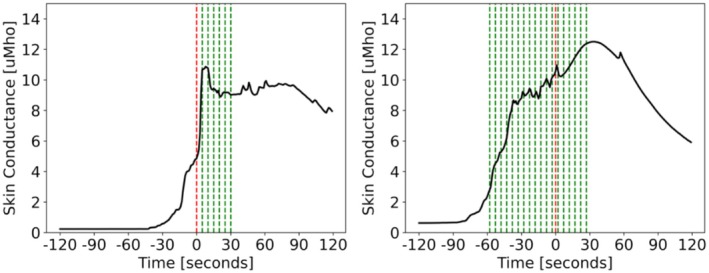
Detected (left) versus predicted (right) HF events. Red dashed line at *t* = 0 indicates expert‐identified HF onset. Green dashed lines indicate the identified HF events by a model. Identification latency is +5 and −58 s for the detected event on the left and predicted event on the right, respectively.

Figure 6 shows detected and predicted HF events. Detected events were identified as events that occurred up to 30 s after HF onset. Predicted events were identified as events that occurred within 60 s before HF onset. Although the model could identify multiple events, only the first event was counted and used to measure the latency.

### 3.6 Model Performance

Table 3 compares the performance of the seven novel models developed in this study on the test data, test data only with typical HF events, and test data with only atypical HF events.

**TABLE 3:** Model Performance—(top) all 10 participants in the Test Set, (middle) 5 participants with typical HF patterns, (bottom) 5 participants with atypical HF patterns. Data are mean ± SD.

Model	Test set								
	Spec (%)	Sens (%)	Pred (%)	F1‐Score (%)	NPV (%)	PPV (%)	Pred rate (%)	Iden rate (%)	Iden latency (second)
Model‐msi	96.0 ± 3.1	29.7 ± 6.9	22.1 ± 5.7	25.3 ± 15.2	98.2 ± 0.9	30.0 ± 28.4	74.6 ± 18.1	89.7 ± 13.3	−20.1 ± 4.5
Model‐d1‐min	98.5 ± 1.7	9.5 ± 6.1	3.2 ± 1.9	12.6 ± 9.1	97.7 ± 1.1	36.8 ± 35.6	12.4 ± 6.1	49.4 ± 27.2	3.2 ± 8.8
Model‐d1‐max	98.2 ± 1.8	33.6 ± 12.8	17.3 ± 6.3	36.1 ± 18.8	98.3 ± 0.8	46.6 ± 33.5	63.1 ± 23.4	78.6 ± 22.0	−13.4 ± 4.9
Model‐d2‐min	98.2 ± 1.8	20.3 ± 8.4	8.1 ± 3.0	23.6 ± 13.0	98.0 ± 1.0	39.6 ± 33.8	33.3 ± 8.9	74.8 ± 25.1	−4.4 ± 4.6
Model‐d2‐max	98.1 ± 1.7	30.8 ± 12.4	18.7 ± 6.9	33.1 ± 18.0	98.3 ± 0.8	42.7 ± 31.5	69.3 ± 22.2	82.8 ± 17.9	−17.6 ± 4.9
Model‐d1d2‐min	98.1 ± 2.0	19.1 ± 7.5	7.3 ± 2.9	22.3 ± 12.2	97.9 ± 1.0	39.3 ± 34.2	25.6 ± 7.0	69.5 ± 26.2	−0.9 ± 6.2
Model‐d1d2‐max	98.4 ± 1.6	31.0 ± 12.7	17.1 ± 6.7	34.6 ± 18.3	98.3 ± 0.8	46.2 ± 32.9	64.2 ± 23.4	79.4 ± 20.5	−14.4 ± 5.6
*Test set only with typical HF patterns*									
Model‐msi	98.4 ± 1.1	34.4 ± 0.9	25.6 ± 2.1	39.5 ± 6.7	97.9 ± 0.8	52.8 ± 23.9	86.8 ± 7.8	98.6 ± 1.8	−20.2 ± 2.1
Model‐d1‐min	98.4 ± 1.3	21.5 ± 7.1	8.9 ± 4.5	25.4 ± 5.0	97.5 ± 0.9	51.0 ± 30.7	31.4 ± 11.6	85.9 ± 11.9	2.1 ± 7.6
Model‐d1‐max	98.8 ± 0.9	50.2 ± 2.3	29.1 ± 1.4	55.2 ± 6.2	98.4 ± 0.6	65.9 ± 21.3	84.6 ± 8.3	95.5 ± 5.1	−18.8 ± 1.1
Model‐d2‐min	98.3 ± 1.2	35.8 ± 3.2	17.5 ± 2.6	40.0 ± 5.2	97.9 ± 0.8	53.7 ± 24.6	58.2 ± 5.9	97.5 ± 2.3	−11.4 ± 3.4
Model‐d2‐max	98.0 ± 1.4	56.0 ± 1.6	36.9 ± 2.1	53.7 ± 9.1	98.6 ± 0.6	55.3 ± 19.9	93.6 ± 3.5	98.8 ± 1.7	−25.7 ± 2.5
Model‐d1d2‐min	98.1 ± 1.4	31.7 ± 4.5	14.5 ± 4.0	35.0 ± 4.1	97.8 ± 0.9	50.4 ± 26.4	47.4 ± 7.3	94.0 ± 5.2	−4.5 ± 4.4
Model‐d1d2‐max	98.1 ± 1.4	54.9 ± 1.7	35.1 ± 1.8	54.2 ± 9.4	98.5 ± 0.6	58.4 ± 22.6	91.7 ± 4.0	97.4 ± 2.3	−24.0 ± 2.1
*Test set only with atypical HF patterns*									
Model‐msi	93.5 ± 2.5	25.1 ± 7.2	18.6 ± 6.0	11.1 ± 3.9	98.4 ± 0.8	7.3 ± 2.9	62.3 ± 17.1	80.9 ± 13.9	−20.1 ± 6.0
Model‐d1‐min	98.9 ± 0.9	4.3 ± 4.3	1.3 ± 1.0	5.6 ± 6.0	98.1 ± 1.0	8.9 ± 9.4	5.4 ± 3.9	25.0 ± 23.3	3.9 ± 7.3
Model‐d1‐max	98.6 ± 0.9	17.2 ± 13.0	7.9 ± 5.4	17.9 ± 12.8	98.3 ± 0.9	20.6 ± 12.9	36.2 ± 24.4	53.5 ± 28.4	−7.8 ± 6.5
Model‐d2‐min	98.2 ± 1.2	11.6 ± 8.5	4.0 ± 2.6	11.7 ± 8.9	98.2 ± 1.0	12.7 ± 9.9	16.9 ± 8.7	46.8 ± 28.8	−2.2 ± 6.7
Model‐d2‐max	98.7 ± 0.8	13.8 ± 10.0	7.8 ± 4.6	15.2 ± 11.0	98.3 ± 1.0	18.0 ± 12.8	40.7 ± 22.0	58.5 ± 22.5	−11.6 ± 5.8
Model‐d1d2‐min	98.7 ± 0.9	8.8 ± 7.1	3.2 ± 2.7	10.0 ± 8.3	98.2 ± 1.0	12.9 ± 10.7	10.9 ± 6.5	37.0 ± 29.9	−0.4 ± 5.1
Model‐d1d2‐max	98.1 ± 1.1	19.3 ± 12.8	9.9 ± 5.7	18.1 ± 11.7	98.4 ± 0.9	18.0 ± 11.2	43.9 ± 21.6	62.8 ± 21.6	−10.6 ± 6.8

## 4 Discussion

This study aimed to develop new methods to predict HF occurrences before subjective perception. We used machine learning methods to train models to identify characteristics of SC signals from a lateral torso worn monitor before the onset of HF events. Multiple models identified HF event onsets prior to subjective perception, defined as HF prediction. The results demonstrate the feasibility of HF prediction, allowing for the opportunity to develop just‐in‐time interventions to mitigate the disruptive effect of HFs.

In order to evaluate the predictive capability of models, new performance criteria were defined (predictivity, prediction rate, identification rate, and identification latency). These criteria were previously defined and used for the delivery of a just‐in‐time intervention (Naghavi and Wade 2022). To correctly interpret model performance, special consideration must be given to what each performance metric means in the context of delivering a just‐in‐time intervention for HF management. Among the performance criteria, Spec and PPV illustrate the false‐positive rate: the higher the Spec and PPV, the lower the rate of false positives. A relatively low false‐positive rate is critical for automatic just‐in‐time intervention systems as higher false positive rates may result in the deployment of interventions that interfere with activities of daily living, generate intervention fatigue, and reduce user acceptance. This is of particular concern in applications like HF management where the number of HF and non‐HF events is not balanced; that is, the number and duration of HF occurrences are substantially lower than non‐HF episodes. On the other hand, higher Pred Rate and Iden Rate illustrate a higher capability to predict (identify before subjective perception) and detect (identify after subjective perception) HF event onsets. Also, Iden Latency demonstrates the average lead or lag time in the identification of the events: lower (more negative) values indicate more capability in predicting events, thus the intervention can be provided seconds before the subjective perception of the event.

Table 3 compares the performances of the seven new models developed in this study. *Model‐msi* shows the best Pred Rate, Iden Rate, and Iden Latency in the test set; however, this comes at the cost of higher false positives (specificity 96% and PPV 30%). The results suggest that this model can predict 74% of events up to 60 s before subjective perception and detect another 15% (Iden Rate—Pred Rate) of events within 30 s after the event onset. This model can identify the event onsets, on average, 20 s before subjective perception.

The other six models using curvefit features show improved Spec and PPV (lower false positive rate), but slightly lower capability for event identification (lower Pred Rate and Iden Rate). However, as 2% improvements in false positive rate are considered significant for a just‐in‐time intervention application (Naghavi and Wade 2022), these six models are preferred. The best performing model is then considered *model‐d2‐max* with the lowest false positive rate (Spec 98%), highest Pred Rate (69%) and Iden Rate (82%), and lowest Iden Latency (−17 s).

In order to benchmark the model performance compared with state‐of‐the‐art methods, we ran the same analysis with two other methods: Freedman (2014) and Forouzanfar et al. (2018). The gold standard method for the number of HF events is the Freedman criteria; that is, > 2 μmho change in sternal SC signal within 30 s. This method had shown high agreement with subjective HF event marks: 95% and 86% of identified HF events were accompanied by a subjective marker during laboratory and ambulatory monitoring sessions, respectively, while all markers were accompanied by a > 2 μmho change in sternal SC signal within 30 s (Freedman 1989). However, this method applies a single threshold on sternal SC signal to identify HF events across all women; thus, this model fails to accommodate between‐subject variability in SC patterns due to HF events. In this study, the Freedman method was tested on the same data and showed comparable Spec (98.2% ± 2.7%), PPV (45.7% ± 36.3%), and NPV (98.3% ± 1.0%) with the best performing model (*model‐d2‐max*), but lower Pred Rate (33.6% ± 21.4%) and Iden Rate (70.7% ± 33.1%). The Iden Latency was also higher (−4 vs. −17 s) indicating that the Freedman method may not be effective at predicting HF onset; thus, it may not be suitable for an automatic intervention system.

Forouzanfar et al. (2018) improved the conventional criterion by implementing more steps in analyzing the SC signal. These steps include conditions based on the SC signal amplitude and rate of change within 30 s before and after an identified HF onset to reject misidentified events (false positives). Since data after an identified event onset were included in the analysis, this algorithm is not suitable for a just‐in‐time intervention application. However, as a reference for comparison, the algorithm was tested on the data collected in this study and achieved Sens = 3.5%, Spec = 99.9% ± 0.0%, PPV = 57.7% ± 35.7%, NPV = 97.8% ± 1.3%, Pred Rate = 21.8% ± 15.8%, and Iden Rate = 63.5% ± 31.7%. The additional steps in Forouzanfar's method compared with Freedman's improved the false positive rate (as improved Spec and PPV) but, on the other hand, impaired the algorithm's ability to predict the HF events (~10% drop in Pred Rate and Iden Rate).

The comparison of results from this study and those from the two reference methods confirmed that the models developed in this work are a significant improvement in the state of the art for HF prediction.

The PPV for the best performing model (*model‐d2‐max*) is less than 42%, meaning that if the algorithm identifies a HF event, it is 42% likely that a HF event has already occurred (in the last 30 s) or will occur (in the next 60 s). All the real‐time models benchmarked in this paper showed a PPV below 50%. Since this is the first study to assess the feasibility of predicting HF, there is no threshold for user acceptability of PPV. The authors hypothesize that the acceptable PPV for HF prediction will depend on the just‐in‐time intervention being delivered. Provision of a cooling intervention that does not accompany a HF event may not be bothersome to the user, given the benefit of receiving automatic interventions. However, this needs to be further assessed in user acceptance testing.

New advances in digital health technologies in the form of wearable devices enable provision of interventions that may reduce the impact of HFs (Composto et al. 2021; Peeke et al. 2024; Wang et al. 2020). Such technologies can benefit from algorithms developed in this study to close the loop by automatically identifying HF events, which may reduce the burden for users and potentially improve user acceptance. The methods developed in this study can predict HF events seconds before subjective perception and, if used to trigger a meaningful intervention, can potentially reduce the severity of the symptoms or even prevent symptoms from happening.

The impact of individual variability on the outcomes of our model will require additional testing. Limitations to this study include the lack of diversity in the study population. HF severity and impact have been shown to vary by a variety of factors including race/ethnicity, age, socioeconomic status, and body mass index, as well as perceived stress and the presence of depressive/anxiety symptoms (Avis et al. 2015; Gold et al. 2006; Thurston and Joffe 2011). Lower sensitivity of other models that employed sternal SC for HF detection was observed in women with higher BMI or who reported high state anxiety at the time of testing (Thurston et al. 2011). Finally, although the majority of women experience sweating with HFs, some experience HFs with heat but little measurable sweating or sweating in places where it is not feasible to use a monitor (Freedman 2014). Finally, the relevance of identifying hot flashes using the method in this report and its potential applications have not yet been evaluated.

Another limitation includes removal of participant‐reported HF events that do not accompany a rise in SC signal. Although previously implemented in studies on objective markers of HFs (Carpenter et al. 2004; Thurston et al. 2011, 2009), this method does not allow investigation of model performance on such events that could have been true experienced HFs. This warrants future investigations on model performance only on subjective reports regardless of the presence of the physiological response. Future studies should include measures to ensure accurate subjective reports of HF events; for example, use of two digital markers.

## 5 Conclusion and Future Work

This study demonstrated the feasibility of predicting HF event onsets before subjective perception using SC signal. The seven novel features developed in this study characterize the early rise in SC signal due to sweat preceding or followed by the HF event onsets. In this study, new criteria for evaluating model performance were introduced. These criteria measure the capability of the system in prediction and detection of the HF onsets. The performance of new models developed in this study was compared with two reference methods, and the results suggest the superiority of the new features in predicting HF onsets before subjective perception.

The authors hypothesize that combining multiple features of SC as well as data from other physiological sensors (e.g., actigraphy, skin temperature, core body temperature, and heart rate) can further improve model performance. Future efforts will focus on developing multivariate models and comparing their performance with univariate models. Follow‐on research will investigate the real‐world use of the algorithms and user acceptance of the just‐in‐time interventions. Early identification of HFs using objective measures is the first step to enabling automatic delivery of just‐in‐time interventions such as the Embr Wave wearable thermal device (Wang et al. 2020), which has been shown to reduce the negative impact of HFs in women and men (Composto et al. 2021; Peeke et al. 2024). This novel technology will enable the first investigation of the relevance of objective HF identification, in addition to the feasibility and efficacy of automatic delivery of HF intervention before subjective perception of a HF. Automatic delivery of HF intervention may have a substantial benefit for reducing the negative impact of HFs and improve quality of life and sleep for millions of individuals.

## Author Contributions

**Nader Naghavi:** data curation, formal analysis, methodology, project administration, software, validation, visualization, writing – original draft, writing – review and editing. **Thomas Cook:** formal analysis, methodology, software, validation, visualization, writing – review and editing. **Ryan Turner:** formal analysis, validation, visualization, writing – review and editing. **Sofiya Shreyer:** data curation, writing – review and editing. **Katherine Colfer:** data curation, project administration, writing – review and editing. **Sonja Billes:** formal analysis, supervision, writing – original draft, writing – review and editing. **Matthew Smith:** conceptualization, funding acquisition, investigation, methodology, project administration, resources, supervision, writing – original draft, writing – review and editing. **Michael Busa:** conceptualization, formal analysis, funding acquisition, investigation, methodology, project administration, resources, software, supervision, writing – review and editing.

## Conflicts of Interest

Nader Naghavi and Sonja Billes are former employees of Embr Labs. Ryan Turner and Sofiya Shreyer received consulting fees from Embr Labs. Matthew Smith is a current employee of Embr Labs.

## Data Availability

Data and code are available from the corresponding author upon reasonable request.
